# Defunctioning Ileostomy Reversal Rates and Reasons for Delayed Reversal: Does Delay Impact on Complications of Ileostomy Reversal? A Study of 170 Defunctioning Ileostomies

**DOI:** 10.14740/jocmr2150w

**Published:** 2015-07-24

**Authors:** Peter Waterland, Kolitha Goonetilleke, David N. Naumann, Mathew Sutcliff, Faris Soliman

**Affiliations:** a Department of Colorectal Surgery, Worcester Royal Hospital, Charles Hastings Way, Worcester WR5 1DD, UK; b Department of General Surgery, Good Hope Hospital, Rectory Road, Sutton Coldfield, West Midlands B75 7RR, UK

**Keywords:** Ileostomy, Defunctioning, Anterior resection

## Abstract

**Background:**

Temporary defunctioning ileostomy can reduce the consequences of anastomotic leak following low anterior resection. However, some patients never have their ileostomy reversed and in other cases the time to reversal of ileostomy can be delayed. The aim of this study was to identify the ileostomy closure rate following anterior resection, time to closure of ileostomy, reasons for delay in reversal and whether delay was associated with an increased complication rate.

**Methods:**

Data were collected retrospectively on consecutive patients undergoing defunctioning ileostomy following anterior resection for rectal cancer, between January 2009 and August 2013. Data were collected on reversal of ileostomy rates, time to reversal, reasons for delayed reversal (defined as > 6 months) and complications following reversal.

**Results:**

One hundred seventy patients were studied (median age 69 years, range 41 - 90 years), of whom 117 (69%) were male. One hundred twenty-seven (75%) patients had their ileostomies reversed. Median time to reversal was 6 months (range 1 - 42). In 63 patients who had delayed reversal, reasons were adjuvant chemotherapy (22, 35%), medical illness (14, 22%), anastomotic leak (9, 14%), and others (4, 7%). Postoperative complications occurred in 33 patients (26%). There was no postoperative mortality. Univariate analysis showed that delayed reversal was associated with an increased rate of complications and longer length of hospital stay following reversal (P < 0.05).

**Conclusions:**

One in four defunctioning ileostomies are not closed following anterior resection in our unit. Of those that are closed, approximately 50% have delayed closure beyond 6 months which is associated with increased risk of complications following their ileostomy reversal.

## Introduction

Anastomotic leak following anterior resection for rectal cancer may lead to increased rate of emergency re-operation, radiological drainage, and increased length of stay in hospital, as well as increased mortality. Formation of a defunctioning loop ileostomy following resection for rectal cancer has been recommended because it may help to reduce the rates of clinically relevant anastomotic leaks, severity of leaks as well as re-operations related to leakage [1-5]. Because of these perceived benefits, the majority of patients undergoing low anterior resections for rectal cancer in modern surgical practice receive defunctioning ileostomies [6]. Such ileostomies are normally intended to be temporary.

Despite the potential benefits, defunctioning ileostomies carry their own burden of morbidity, with up to two-thirds of patients having stoma related morbidity [7], as well as negative quality of life effects [8, 9]. Some authors have therefore recommended that stoma time be kept to a minimum [7]. Earlier ileostomy closure may also reduce postoperative nausea and vomiting [10]. However there is also risk of morbidity following subsequent stoma reversal [11, 12], in particular a risk of surgical site infection (SSI) [13]. A balance must therefore be struck between whether a stoma should be fashioned, given the risks of subsequent surgery, and if it is to be reversed, the timing of such surgery. The aim of the present study was to identify the ileostomy closure rate following anterior resection for rectal cancer at a UK NHS Trust, including time to closure of ileostomy, reasons for delay in reversal, and whether delay was associated with an increased morbidity. Such information may help to guide decision making for patients and surgeons alike in this delicate balance of surgical risks and benefits.

## Methods

### Patient selection and setting

Unique patient identification details were obtained from the hospital episode statistics (HES) of our NHS Foundation Trust for all consecutive patients who underwent ileostomy during primary surgery that was coded as anterior resection for rectal cancer (code H33.4) from January 2009 to August 2013. The NHS Trust consists of three geographically separated hospitals within the West Midlands, UK. These patients were selected because defunctioning ileostomy is usually fashioned with the intention to reverse the ileostomy at a future date [14].

### Data collection

Information was obtained regarding each patient’s operation and subsequent clinical progress from electronic medical records as well as case records and clinic letters. Data obtained included patient demographic details (age and sex), stage of disease, type of operation, as well as details regarding reversal of ileostomy, time to reversal, and reasons for delayed reversal (defined as > 6 months). The primary outcome recorded was postoperative complication within 30 days of reversal of ileostomy. The secondary outcome was length of hospital stay.

### Definitions

Delay in ileostomy reversal was defined as being more than 6 months from the original defunctioning loop ileostomy procedure. Histological staging of tumors was undertaken by consultant histopathologists by examining the surgical specimens, and was classified according to the Dukes classification of colorectal cancer.

### Statistical analysis

Categorical data were compared using Chi-squared statistical analysis. Univariate Cox regression analysis was used to examine the relationship between individual patient factors and delay in reversal of ileostomy, and whether delay was related to adverse outcomes (i.e. the presence of complications).

## Results

### Demographics

During the study period 170 patients underwent defunctioning ileostomy following anterior resection for sigmoid or rectal cancer. The median age (range) was 69 years (41 - 90 years). Of these patients, 117/170 (68.8%) were male.

### Staging

Tumor staging data were available for all patients. These included Dukes A in 49/170 (28.8%), Dukes B in 57/170 (33.7%), Dukes C in 48/170 (27.9%), and Dukes D in 16/170 (9.6%) (Table 1).

**Table 1:** Patient Characteristics and Dukes Classification

	Dukes classification			
	A (n = 49)	B (n = 57)	C (n = 48)	D (n = 16)
Median age, years	69	70	68	67
Male, n (%)	29 (59)	46 (80)	34 (71)	8 (50)
Ileostomies reversed, n (%)	47 (96)	41 (72)	32 (67)	7 (44)

### Reversal of ileostomy

Of the 170 patients who had defunctioning ileostomies, 127 (75%) underwent subsequent reversal of ileostomy. Median time to reversal was 6 months (range: 1 - 42 months). Of these ileostomy reversals, 63/127 (50%) were delayed longer than 6 months. The reasons for delay were due to adjuvant chemotherapy in 22/63 (35%), surgical complications in 13/63 (21%) which included anastomotic leak following the initial operation in 9/63 (14%), adhesive small bowel obstruction in 3/63 (5%), and anastomotic stricture in 1/63 (2%) patient. Medical complications were seen in 14/63 (22%), which included eight patients with acute kidney injury due to high output stoma (Table 2).

**Table 2:** Reasons for Delayed Reversal of Ileostomy

Postoperative adjuvant chemotherapy	35% (22/63)
No health-related reason identified	22% (14/63)
Non-surgical complications CABG (n = 1) PE (n = 1) Renal failure (n = 8) Prolonged postoperative fatigue (n = 2) Patient wishes (n = 2)	22% (14/63)
Surgical complications Symptomatic anastomotic leakage 14% (9/63) Adhesive SBO 5% (3/63) Anastomotic stricture 2% (1/63)	21% (13/63)

When comparing the delayed and non-delayed groups, there were no statistical differences in number of patients or age between the groups. Patients with Dukes A tumor were statistically more likely to be in the non-delayed group, and those with Dukes C and D tumors were more likely to be in the delayed group (Table 3).

**Table 3:** Patient Characteristics and Timing of Reversal of Ileostomy

	Timing of ileostomy reversal		
	Non delayed	Delayed	P value
Total number, N (%)	64 (50.3%)	63 (49.7%)	NS
Male, n (%)	38 (46.3%)	44 (53.7%)	NS
Female, n (%)	31 (68.8%)	14 (31.2%)	0.05
Mean age, years	69	67	NS
Median length of inpatient stay			
> 4 days	22	32	< 0.05
< 4 days	44	25	< 0.05
Complications, n (%)	11 (33.3)	22 (66.7)	< 0.05
Dukes stage, n (%)			
A	35 (81.3)	8 (18.7)	< 0.05
B	20 (48.7)	21 (51.3)	NS
C	8 (25.8)	23 (74.2)	< 0.05
D	3 (42.8)	4 (57.2)	

### Postoperative outcomes

The median length of inpatient hospital stay following reversal of ileostomy was 4 days (range: 1 - 62 days). Postoperative complications occurred in 33/127 (26%) patients. There was a statistically significant rate of complications following reversal and a median length of hospital stay following reversal of > 4 days in the delayed group. A further 5/127 (3.9%) patients were re-admitted within 30 days for postoperative complications. There was no postoperative mortality.

### Reasons for stoma non-reversal

The following causes were associated with having a permanent stoma: cancer stage IV 23% (10/43), patient refusal 14% (6/43), patient death prior to reversal 11% (5/43) and other causes 21% (9/43) (duodenal cancer (n = 1), pelvic recurrence (n = 2), High risk (n = 2), refashioning of ileostomy (n = 1), rectovaginal fistula (n = 1), and medical illness (n = 2)).

Comparison between patients in the reversed (R) and non-reversed (NR) group revealed a higher anastomotic leak rate 16% (NR) vs. 7% (R) and stage IV disease 44% (NR) vs. 30% (R) which was found to be significant (P < 0.001).

At the end of the follow-up period, there were 116/170 (69%) patients alive without a stoma and 30/170 (17%) alive with a stoma. Twelve of 170 (7%) patients died without a stoma and 12/170 (7%) died with a stoma in the series.

### Preoperative neo-adjuvant treatment

Seventy-two (42%) patients received neo-adjuvant treatment. Forty-five had short course radiotherapy (SC) and 27 had long course chemoradiotherapy (LCR). In the stoma reversed group there were 39 SC and 18 LCR and in the non-reversed group six SC and nine LCR. There were 17 SC and eight LCR in early stoma reversal group compared to 22 SC and 10 LCR in delayed group (Table 4).

**Table 4:** Neo-adjuvant Treatment and Timing of Reversal of Ileostomy

	Short course radiotherapy	Long course radiotherapy
Stoma reversed	39	18
Stoma non-reversed	6	9
Delayed stoma reversal (> 6 months)	22	10
Early stoma reversal (< 6 months)	17	8

There were 13 complications in the neo-adjuvant group (four in LCR and nine in SCR) compared to 23 in the non-neo-adjuvant group. There was no correlation to pre-operative neo-adjuvant treatment and complications following ileostomy reversal.

## Discussion

The major finding in this study was that reversal of ileostomy in patients having delayed closure is associated with increased risk of complications and increased length of hospital stay. Of those that are closed, approximately 50% have delayed closure > 6 months following their ileostomy formation. Also, one in four defunctioning ileostomies are not closed following anterior resection for rectal cancer in our unit. In 50% of the patients, the delay could be explained by postoperative adjuvant chemotherapy, non-surgical complication and symptomatic anastomotic leakage and small bowel obstruction. Adjuvant postoperative chemotherapy has been proposed as an important reason for delaying stoma reversal [15, 16]. In the present study population, postoperative adjuvant chemotherapy contributed to a delayed stoma reversal in more than a third of patients having delayed closure. The available literature is unclear about the optimal timing for reversal of defunctioning ileostomies, but generally reversal is recommended within 8 - 12 weeks following low anterior resection. The rationale behind this recommendation is to obtain adequate healing while avoiding a prolonged presence of a defunctioning ileostomy with subsequent discomfort for the patient, risk of stoma-related complications such as dehydration, and also this allows the patient sufficient time for recovery from the initial resection, softening of intra-abdominal adhesions and resolution of inflammation and edema within the abdomen and around the stoma orifice. However, a recent randomized controlled trial concluded that early stoma closure is feasible in selected patients, with reduced hospital stay, bowel obstruction and medical complications [17]. It is not known how often defunctioning ileostomies are reversed after 8 - 12 weeks in routine clinical practice as studies in population-based settings are rarely described in the surgical literature. Twenty-two percent of the patients in this study experienced a delayed stoma reversal without any evident health-related reason for the delay. We conclude that this was due to the fact that stoma reversal was given lower priority when competing with other benign conditions requiring surgery.

There was a statistically significant rate of complications following reversal in the delayed group. A recent review published from a Danish group also revealed similar findings to those who had delayed ileostomy closure. They reported a significant association between the delayed ileostomy closure and complications after low anterior resections, 51 (59.3%) versus 27 (37%) (P = 0.005) [18]. One of the possible reasons for more complications in the delayed group could be attributed to the adjuvant chemotherapy in our study.

Rates of stoma closure amongst patients with defunctioning ileostomies following anterior resection have been variably reported, from 68% to 75.1% [14, 15], and as high as 91.5% in one report [19]. Our study population demonstrates 75.7% reversal rate, which is within this range.

Of all defunctioning ileostomies in this study, 25% were never reversed and subsequently became permanent stomas. The most important reason for a defunctioning ileostomy to become permanent in the present study population was stage IV cancer. Another major reason for not reversing stomas was symptomatic anastomotic leakage following anterior resection. Results from recently published large studies have demonstrated that there is a risk between 18% and 25% for defunctioning ileostomies to become permanent [14, 20-22]. Symptomatic anastomotic leakage [14, 20-22] is identified as independent risk factors for permanent stoma, and the results of the present study are in line with these findings.

National Bowel Cancer Audit Project (NBOCAAP) 2013 report revealed that nearly one-third of anterior resection patients would be with a stoma at 18 months follow-up. There data support patient counselling for a temporary ileostomy should include a non-closure rate of 40%, a median closure delay of 7 months for those closed, and an approximately 10% chance of death with a non-reversed intestinal stoma at 18 months [23].

The present study of closure of defunctioning ileostomies is based on a homogenous group of patients having a defunctioning loop ileostomy after an anterior resection for rectal cancer. The main conclusion of this study is the increased rate of complications following a delayed loop ileostomy closure. Large randomized controlled trials are necessary to evaluate the correlation between complications and the timing of loop ileostomy closure.
